# Research on Voluntary Carbon Information Disclosure Mechanism of Enterprises from the Perspective of Stakeholders—A Case Study on the Automobile Manufacturing Industry

**DOI:** 10.3390/ijerph192417053

**Published:** 2022-12-19

**Authors:** Chensi Guo, Wenyan Pan

**Affiliations:** School of Safety Science and Emergency Management, Wuhan University of Technology, Wuhan 430070, China

**Keywords:** stakeholders, corporate disclosure, evaluation methods, facilitating factors, inhibiting factors

## Abstract

As the primary source of carbon emissions, enterprises must work hard to save energy, reduce emissions, and disclose timely carbon information to the public. As a key means of communicating carbon management performance to stakeholders, carbon information disclosure is directly tied to the future sustainability of enterprises. Based on panel data of 118 listed firms in the automotive manufacturing industry from 2017 to 2021, this study rates the sample companies’ quality of carbon information disclosure. The impact of the government, creditors, media, employees, and suppliers on such disclosure is also examined from the stakeholders’ standpoint. The findings reveal that: (1) Although there has been a gradual increase in the degree of disclosure, overall levels are still low, and the willingness to voluntarily disclose is insufficient. (2) When other variables are neglected, the government, creditors, media, and employees all assist enterprises in disclosing carbon information, but the influence of suppliers will inhibit such disclosure. In the context of a complex economic system, the level of carbon disclosure is positively correlated with the government, the media, and employees, while negatively correlated with creditors. The influence of suppliers is not significant. These findings may aid in formulating related policies from different dimensions, directing enterprises to publish carbon information actively and strengthening carbon management.

## 1. Introduction

On 11 March 2021, the 13th National People’s Congress (NPC) agreed to adopt the resolution on the 14th Five-Year Plan and the general framework of the 2035 Visionary Goals. The meeting incorporated carbon peaking and carbon neutrality into the development goals, referred to as the “double carbon” strategy. During the 14th Five-Year Plan, the national carbon market will encompass seven high-emission industries, including petrochemicals, iron and steel, and chemicals. China will gradually form the world’s largest carbon market with more than 7000 emission control entities and a total annual CO_2_ emission scale of 4–5 billion tonnes. China has actively addressed climate change and implemented several policy changes. At the macro level, China has advanced its energy structure adjustment and vigorously carried out energy conservation, carbon reduction, and ecological construction. At the micro level, China has established a greenhouse gas emission accounting, reporting, verification, and monitoring system for key emission units in eight key industries [1], and encouragement of the development of a carbon trading market [2].

Carbon disclosure is interesting to regulators and researchers for several reasons. Firstly, the general public and potential investors are paying attention to climate change. A range of climate governance policies means that companies face specific regulatory risks. As a result, businesses view climate change as a significant issue. The stock market is also becoming more interested in carbon information disclosure [3]. Secondly, China has intensified the promotion and development of the carbon emission trading market to better assist major companies in setting emissions limits [4]. A more vibrant carbon trading market is supported by improving the quality of carbon disclosure [5]. Finally, scholars have embraced the idea of bringing direct ecological implications into the study of commercial organizations. Carbon disclosure has become a key method for strengthening climate change resilience, boosting the use of renewable energy, and fostering sound external accountability.

Academics have conducted several types of research on the factors influencing carbon information disclosure and disclosure impacts. These influencing factors include country-related factors and company-related factors. At the macro level, enterprises from common law nations and nations with tougher restrictions are more inclined to participate in CDP projects [6]. The level of rigor of government regulations has a big impact on carbon information disclosure [7]. Social and financial markets are also key determinants of such disclosures [8]. Damert et al. compared data from 45 top businesses in the worldwide steel sector between 2008 and 2013. The experiment’s findings show that institutional variables can have a favorable impact on businesses’ efforts to reduce their emissions [9]. At the micro level of the enterprise, mixed results have been reported by studies on the influence of business political affiliation on carbon disclosure, ranging among positive correlations, negative correlations, and non-linear relationships [10,11,12,13]. Other important corporate carbon disclosure participation determinants include company size, environmental laws, and legal standing. Moreover, the frequency and timeliness of reporting to the board of directors are crucial for improving carbon disclosure and carbon performance [14].

Although numerous studies have demonstrated that environmental information disclosure considerably and favorably affects business economic performance, carbon disclosure’s impact is unclear [15]. Some studies believe that carbon emissions disclosure positively influences company value [16]. In addition, Alsaifi et al. report that investors react negatively to carbon disclosure announcements made by FTSE 350 companies through the CDP [17]. However, there are other economic consequences of disclosing carbon information. Companies gain more than they might anticipate from carbon information measurement and publication [18]. For instance, effective carbon disclosure can assist businesses in managing their financial risk [19]. Carbon disclosure will considerably lower the cost of corporate borrowing for companies with subpar carbon performance [20].

Enterprises, as the basic production unit of society, are not only the driving force of economic development but also the primary source of greenhouse gas emissions. They are responsible for making high-quality disclosure to help the success of national emission reduction actions. In order to increase enterprise enthusiasm to engage in disclosure, it is essential to understand the motivations behind it and to develop relevant measures. Early studies on the disclosure of carbon information concentrated on an organization’s internal traits, like size and profitability. However, as research progressed, the perspective gradually shifted to the external aspects, i.e., how the external factors of enterprises affect carbon information disclosure. However, the effect on stakeholders is still too uniform. Stakeholder theory emphasizes, in particular, that the various interests and objectives associated with a company shape the company’s management strategy. Carbon disclosure can be seen as a stakeholder demand for information when responding to pressure from climate change issues, and the response of companies is to provide carbon emissions information. Researchers looked into the greenhouse gas emissions of 431 European businesses. They found that stakeholder pressure from outside sources significantly affected greenhouse gas emissions [21]. For example, creditor pressure is significantly correlated with corporate carbon disclosure [22]. Media attention can motivate companies to actively disclose environmentally relevant information [23]. Furthermore, business climate change disclosure is positively connected with the impact of large institutional investors [24]. Similarly, internal company stakeholders, such as a board of directors composition, can have a favorable impact on corporate carbon performance and disclosure [25]. However, most research looks at specific stakeholders as one of the contributing elements. Only a few articles have looked at the stakeholder perspective. Users of carbon information frequently interact with each other. Establishing indicators to explore the relevance of a particular stakeholder inevitably leads to bias and neglect of more important influencing factors. As stakeholders become increasingly concerned about environmental issues, companies will actively disclose relevant carbon information to the outside world through various channels to better their survival and image. Therefore, the stakeholder theory can assist in illuminating the fundamental reasons for such disclosure. So, this study aims to thoroughly analyze stakeholder influences and empirically assess the main influencing mechanisms and elements. As a result, public policymakers will be better able to design a disclosure environment that will successfully encourage businesses to report their carbon information.

Regarding the scope of research, current studies need more specific research on a single industry. Different industries have different characteristics, which may influence carbon disclosure to varying degrees. Since the reform and opening up, the automobile industry in China has been steadily expanding and developing. It has been instrumental in both national economic development and major social change. The new energy vehicle sector entered a new development phase in 2021, the first year of the 14th Five-Year Plan. New energy vehicles are marked with “energy saving” and “emission reduction”. Since 2010, major Chinese automakers have been introducing new energy vehicles progressively. As a result, the economy and ecology are greatly impacted by the rise of the automotive sector. Therefore, the research in this paper is based on 118 listed companies in the automotive manufacturing industry. As a premise of the study, there is no accepted measurement system for evaluating such disclosures [26]. The main evaluation techniques now in use are content analysis techniques and CDP project responses. However, because China’s automotive manufacturing companies participate in CDP at a relatively low rate, using the CDP database will result in inaccurate statistics. Consequently, this paper adopts the content analysis method, and provides a carbon information disclosure evaluation system for the automotive manufacturing industry. The research information was manually gathered from primary sources, including annual reports and CSR reports.

This paper makes the following contributions to theory and practice. Firstly, the data are first-hand accounts of the study and were collected for the reporting period ending in 2021. The research data are relatively new and reflect the latest developments. Secondly, a novel quantitative indicator system for corporate carbon information disclosure is provided using a content analysis method. Additionally, it offers an innovative approach for further study. Thirdly, this paper focuses on the mechanism of stakeholder action. The research standpoint is original. The paper constructs a systematic research framework covering government, creditors, media, employees, and suppliers. It goes beyond stakeholders’ traditional single research perspective on environmental information disclosure. By experimentally evaluating each factor’s influence, in the end, the major influencing elements of such disclosure are determined. This might offer a better orientation for the creation of policies. Finally, there has not been much academic discussion of how suppliers and employees affect carbon disclosure. This publication advances this field with the most recent data, validates earlier research, and summarizes it.

## 2. Data and Methods

### 2.1. Research Hypothesis

#### 2.1.1. State

Government departments’ pertinent recommendations greatly influence how much information businesses provide about greenhouse gases [27]. The Chinese government has also been proven to have a favorable and considerable influence on listed companies’ disclosure of environmental information in China [28]. Aside from policy assistance, the Chinese government is actively researching market mechanisms to limit greenhouse gas emissions and has developed several regulatory measures to facilitate the formation of a carbon emissions trading market. As a result, listed firms can be expected to sense increasing government power and interact with the government by actively participating in carbon disclosure measures to reduce policy risks.

According to social capital theory and the government’s supportive hand theory, enterprises want a secure environment for growth and greater resources for development by creating close ties with the government through adopting state equity [29]. When the government becomes the primary investor in a corporation, the company’s political ties become a valuable resource for the company to achieve higher performance, and the government can help the company acquire more favorable policies. Companies that hold state stock send a political signal that the government supports the company and is willing to share the risk. To better meet China’s low-carbon growth target, state-owned companies are typically subject to higher disclosure requirements. Therefore, companies are more proactive in disclosing information about their social responsibility to reflect their environmental contribution and gain more support from their stakeholders. Consequently, we offer the following hypothesis in light of the research above.

**H1:**  *The stronger the government’s influence, the greater the level of carbon disclosure*.

#### 2.1.2. Creditor

Based on the “green finance” policy, corporate finance is influenced by financial and environmental performance. By continuously strengthening the environmental verification process for refinancing, the state has transformed the negative environmental impact of an enterprise’s obsession with financial gain into a constraint on its refinancing. From the creditor’s perspective, environmental infractions will result in higher fines. The corporation might be unable to repay the loan as its exposure grows. The rights of creditors may suffer because of this. As a result, when a company depends on outside investment, it must consider its environmental performance to win over its creditors. Higher debt ratios will induce businesses to divulge more environmental data [30]. This is an important step by the state to improve environmental issues from the perspective of the relationship between enterprises and creditors. Whether for legitimacy proof or policy pressure, enterprises will appropriately improve the quality of their environmental disclosure to demonstrate their environmental friendliness, especially if they have a good corporate governance structure.

Large environmental investments, however, may not always result in loans because of the information asymmetry, and environmental information is not included in the audit’s purview. According to the cost-benefit principle, firms are more inclined to reduce information sharing about the environment to hide their poor environmental behavior, especially those with low financial resources and poor environmental performance. It is, therefore, hard to determine, at the theoretical level, how finance requirements impact the caliber of environmental information sharing. Consequently, we offer the following hypothesis in light of the research above.

**H2a:**  *The greater the influence of creditors, the higher the level of carbon disclosure*.

**H2b:**  *The greater the influence of creditors, the lower the level of carbon disclosure*.

#### 2.1.3. Media

Modern information technology has transformed the media into an external force for corporate monitoring. A rise in environmental information disclosure should coincide with a rise in public interest in environmental issues due to increased media coverage [31]. There is a strong link between media coverage volume and environmental information sharing [32]. Moreover, in particular, the coverage that is non-negative and policy oriented has a major positive impact on how corporations disclose their social responsibility. In this paper, we argue that media publicity coverage can discipline CSR behavior. Increased media coverage will encourage businesses to reveal more carbon information actively. Consequently, we offer the following hypothesis in light of the research above.

**H3:**  *The greater the media attention, the higher the level of corporate carbon information disclosure*.

#### 2.1.4. Employee

The stakeholder theory states that employee information demands greatly influence how much environmental information is disclosed. In addition to serving as internal stakeholders and a source of labor, firm employees also directly participate in manufacturing and operating processes and are harmed by environmental contamination. As environmental awareness grows, employees become more concerned about the organization’s environmental performance. Enterprise employees recognize that negative environmental strategies can lead to negative environmental performance, bring about penalties or damage the image of the enterprise, and even jeopardize employee rights. Employees are especially interested in how the firm views its environmental strategy since employee rights and the company’s prospects are connected. There is no doubt that the requirements of the workforce may also have some bearing on the publication of environmental information. Employees are even more invested as shareholders in companies with a stake in the business. To further ensure that their opinions are heard by the management, employees in large companies frequently organize themselves into trade unions or unique corporate bodies (such as a department specifically dedicated to environmental issues). Due to employee pressure, businesses will actively reveal carbon information [21]. The number of employees significantly improves such disclosure [33]. In addition, employees themselves need more transparent information about the environment so that their rights are not compromised. Consequently, we offer the following hypothesis in light of the research above.

**H4:**  *The greater the internal influence of employees, the higher the level of carbon disclosure*.

#### 2.1.5. Supplier

Looking at the supply chain as a whole, the capital markets and investors are the information’s intended audience when environmental information is provided in a company’s annual report. Environmental data, however, also benefit suppliers. When an organization fails to satisfy the expectations of its suppliers throughout the supply chain, the latter will search for new, more dependable partners or even take action against wrongdoing (e.g., by suspending supplies). All of these outcomes might adversely impact the company’s performance. If companies maintain strong ties with their suppliers, they can optimize the benefits of business financing. Supplier concentration reflects the strength of inter-firm interactions and may have an impact on disclosure [34]. The greater the concentration of suppliers, the more privileged position they hold within the company. In other words, the more pressure the supplier may put on the company. To maintain a positive connection with their suppliers, companies will be more willing to share information to lessen the degree of information asymmetry.

However, information disclosure in the capital market abides by the supply and demand equilibrium law. When there is a high concentration of suppliers, private communication between businesses is more effective and less expensive. As a result, businesses have less motivation to provide information to the public market. Consequently, we offer the following hypothesis in light of the research above.

**H5a:**  *The greater the influence of suppliers in a company’s supply chain, the higher the level of carbon information disclosure*.

**H5b:**  *The greater the influence of suppliers in a company’s supply chain, the lower the level of carbon information disclosure*.

**Figure 1:**
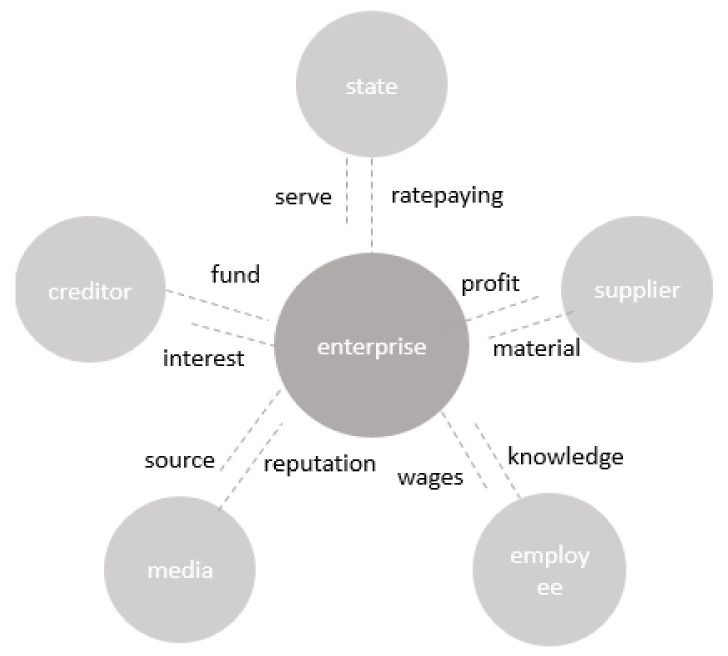
Stakeholder influence mechanisms.

The mechanism of influence between stakeholders and enterprises is shown in Figure 1.

### 2.2. Sample Selection and Data Sources

The study uses 118 publicly traded companies in the automotive manufacturing sector from 2017 to 2021 as its sample. This paper’s data come from the following sources: the financial data and basic information data of the companies are obtained from the CSMAR database; the media attention data are sourced from the CNRDS database; and the carbon information disclosure data are manually collected from the annual reports and CSR reports downloaded from Juchao and Shanghai Stock Exchange. The data were processed using Stata 16.0 and Excel.

### 2.3. Selection of Variables

#### 2.3.1. Explanatory Variables

Most academics opt for the content analysis approach and index method when building carbon information disclosure systems. Notable academics in this regard are Chen Hua [35] and Li Huiyun [36]. In addition to reading a substantial amount of literature to compile the indicators utilized by different experts, this paper summarizes, organizes, and clarifies the carbon information supplied by the sample corporations. Additionally, a number of documents published after the formal start of the carbon market and the most recent instructions released by the Ministry of Ecology and Environment in May 2021 were merged to choose indicators appropriate for the vehicle manufacturing business. Seven major indicators and seventeen minor indicators make up the completed evaluation system. It does not, however, incorporate the use of weights carried out by other academics [36]. Although assigning different weights to various items can give a solid foundation for the index’s correctness, there is no defined formula for assigning weights. Even if some researchers used surveys and other methods to gather a more specialized basis for establishing the index, the results would still be tainted by elements such as the survey method’s randomness, the sample size’s constraints, and the subjectivity of experts. Since it is difficult to predict the variability of the weights for a multi-annual sample, no weights are set here. Instead, the scores of the disclosure items are directly added up and can be used as the average after combining a number of subjective and objective factors. The specific items are set as in Table 1.

The evaluation system has a range of scores [0–34]. In particular, a value of 2 is assigned to a company that has published a social responsibility report and includes a graph, 1 to a text-only description, and 0 to a non-publication. For the following five parts, non-disclosure is rated as 0, basic disclosure is rated as 1, and full disclosure is rated as 2. If the company has completed the IS014001 environmental management system certification, it will be given a value of 1; if it has not, it will be given a value of 0. Similarly, if the company has undergone an inspection by an impartial third party, it will be given a value of 1; and if it has not been certified, it will be given a value of 0.

#### 2.3.2. Control Variables

The following variables were chosen as control variables in the study model based on the research results of international scholars.

(1) Business size (Size)

Due to greater stakeholder demand for information from large enterprises and lower costs for large enterprises to prepare information, the majority of scholars believe that the level of information transparency and firm size have a strong beneficial association. The disclosure of carbon information follows the same logic [37]. The bigger the company, the more attention it receives and the more it has to establish or uphold its reputation through carbon information disclosure [10]. In order to account for its effects, this article substitutes the natural logarithm of total year-end assets for the enterprise size variable.

(2) Profitability (RA)

Less profitable businesses frequently reveal more social responsibility data to enhance their brand. According to certain research, a company’s profitability is inversely connected to the amount of information it discloses about greenhouse gas emissions [38]. Other research has shown that a company’s level of disclosure positively correlates with its financial performance [39]. The greater a company’s return on total assets (ROA), the more carbon accounting information it exposes. Thus, profitability may affect how enterprises disclose their carbon emissions. The paper accounts for this influence using ROA as a stand-in for profitability.

Additionally, this study uses the dummy variable “Industry” to indicate whether a business is the main emission unit because high-carbon emission industries harm the environment more than other businesses do. If the answer is yes, the value is 1, and if it is no, it is 0.

For detailed descriptions and representation symbols for each variable, see Table 2.

### 2.4. Model Construction

The hypotheses in this study were all tested using multiple regressions, and the basic regression models designed are as follows. The relevant explanatory variables are brought into the regression model separately to investigate the fundamental role of stakeholders on corporate carbon information disclosure and further explore their role’s mechanism.

(1) $$CDI_{t}=\alpha +\beta_{1}State_{t}+\beta_{2}creditor_{t}+\beta_{3}Media_{t}+\beta_{4}Staff_{t}+\beta_{5}SR_{t}+\beta_{6}Size_{t}+\beta_{7}RA_{t}+\sum Industry+\varepsilon$$

## 3. Results and Analysis

### 3.1. Descriptive Statistics

Table 3 displays the descriptive statistics for the overall carbon disclosure index. The mean value of carbon information disclosure for the five years is 5.3203, with a minimum of 0 and a maximum of 27. The sample companies’ overall level of disclosure is low, owing to the right-skewed distribution of all sample data and the presence of companies that do not reveal any carbon information. The standard deviation was 5.1491, indicating that the level of disclosure varies relatively widely among enterprises. Further investigation showed that the disclosure index’s mean value was 3.1102 in 2017, 4.8220 in 2018, 5.3898 in 2019, 5.6017 in 2020, and 7.6786 in 2021, showing an upward tendency in the level of carbon information disclosure over time. The standard deviation for 2017, 2018, 2019, 2020, and 2021 were 4.2342, 4.6397, 5.1454, 4.9601, and 5.6627, respectively, showing the variability in the level of carbon information disclosure was also typically growing year by year. Additionally, it was discovered that only a small percentage of non-key emission organizations had voluntarily revealed carbon information. Most companies who made carbon information disclosures were key emission companies. It is evident that voluntary disclosure levels of listed firms need to be increased.

### 3.2. Correlation Analysis

To guarantee that the estimation results for the coefficients of each variable are not seriously biased, the correlation coefficients of all the variables were calculated and analyzed before the multiple regression of the model.

Table 4 displays the correlation matrix for all variables. The data in the table represent Pearson correlation coefficients between two of the variables in the horizontal and vertical columns. The correlation coefficients between each explanatory variable are often less than 0.5. Furthermore, the variance inflation factors are used to diagnose the degree of multicollinearity of the independent variables. The calculated VIFs are all less than 10, this suggests that the correlation between these variables is not high (Table 5). The operation of the model was not easily disrupted by multicollinearity. The control variables chosen for this study were reasonable because there was a strong association between carbon information disclosure and firm nature, size, and profitability. Moreover, government, creditors, media, employees, and suppliers are all significantly tied to carbon information disclosure, which lays a foundation for the subsequent hypothesis testing and relationship analysis. As for their effect, coefficients and significance need to be analyzed with the help of the subsequent multiple regression analysis.

### 3.3. Regression Analysis

Table 6 reports the full sample regression results for each stakeholder and corporate carbon disclosure. The first five columns of the table show the results of the regressions considering only government, creditors, media, employees, and suppliers, respectively. Column (6) presents the regression results when all five stakeholders are considered simultaneously. Column (7) presents the regression results when the five stakeholders and the control variables are considered. The first five columns show that Hypothesis H1, Hypothesis H2a, Hypothesis H3, Hypothesis H4, and Hypothesis H5b are all initially validated. That is, without considering other influencing factors, the government, creditors, media, and employees all have a positive influence on corporate carbon disclosure behavior. At the same time, pressure from suppliers can inhibit such disclosure. Moreover, the regression coefficients are significant at the 1% level. As demonstrated by column (6), when all five stakeholders are taken into account at once, the influence of creditors becomes insignificant. Furthermore, the sign of the supplier changes from negative to positive. Hypothesis H5a is tested. The remaining variables remain to be strongly and favorably related to corporate carbon disclosure. When control variables are included, the overall fit increases to 0.608, as seen in column (7), suggesting that the influencing components with good explanatory power on such disclosure are eventually attained. Compared to the regression results for the influence of a single factor, the explanatory variables remain largely significant in the combined regression results. This indicates that the explanatory variables are robust in both single and complex economic environments. Business nature, size, and profitability all significantly affect disclosure. After controlling for these factors, the government significantly promotes carbon information disclosure at the 10% level. The demand for financing becomes significant at the 1% level to inhibit it, supporting hypothesis H2b. The promotion effect of the media decreases from a 1% to a 5% significance level. The positive influence of employees remains significant at the 1% level. Moreover, the impact of suppliers turns out to be insignificant. In addition, the F-values for the overall test of the equations in columns (6) and (7) are 91.62 and 115.3, respectively, indicating that both regression models pass the overall test.

As can be seen from the above results, in a complex economic and institutional environment, companies’ environmental behavior and attitudes are changing. The outcomes for government, media, and employees are more robust. They continue to exhibit a strong positive association with the degree of carbon information disclosure. It indicates that they are important factors affecting such disclosure, despite external economic and regulatory context changes. It is noteworthy how creditors’ impact on corporate carbon disclosure has changed from a facilitative effect under a simple condition to a discouraging effect under a complicated requirement. The variable “Size” demonstrates that a larger company tends to provide more carbon information than a smaller one. Combining the two various theories, it is possible to suggest that the relationship between carbon information disclosure and creditors is moderated by business size. The change in supplier salience may be that there are better channels for suppliers to obtain information than the open market. First of all, suppliers have many contacts with employees at all levels of the company in their daily business dealings. They have access to a variety of sources of corporate information. Secondly, communication of some important information may be written directly into the contract. This is carried out to make the mutual ties stronger. Finally, to improve communication and oversight, significant suppliers and the company will send each other top management or board members [40]. Through these channels, suppliers have access to more information than open market disclosure, which is more recent and relevant. Consequently, when making carbon disclosures, corporations will first consider the pressure from the government, creditors, the media, and employees. The impact of suppliers is minimal.

### 3.4. Robustness Tests

Robustness tests are also carried out in this article to improve the reliability of the results. Firstly, a 1% shrinkage tail is applied to all continuous variables to account for the impact of extreme values. Repeating the aforementioned multiple regression regressions, as shown in Table 7, yielded results similar to the earlier research. Secondly, referencing relevant research, the natural logarithm of sales revenue takes the place of the company size measurement, and RA is replaced by ROE. Regress the model again, as shown in Table 8, the results of core explanatory variables remain mostly the same.

## 4. Discussion and Conclusions

### 4.1. Conclusions

From a stakeholder viewpoint, this study investigates how internal and external economic and political stakeholders, acting alone or in concert, affect corporate carbon disclosure. This paper first constructs an evaluation system for carbon information disclosure in the automotive manufacturing industry. For 2017–2021, the information for 118 listed companies was manually gathered and compiled. The findings indicate that while there has been a yearly rise in disclosure levels, the overall level is still low. Meanwhile, the level of disclosure varies greatly between enterprises. Secondly, a multi-stakeholder structure (government + creditors + media + employees + suppliers) is built to solve the research topics, focusing on resources and demands from politics and markets. Based on 590 reliable observations, this study confirms that, regardless of the impact of other factors, the government, creditors, media, and employees all positively influence corporate carbon disclosure. However, the influence of suppliers will inhibit such disclosure. When the five stakeholders’ roles are taken into account collectively, the effect of the government, media, and employees remains positive. The influence of creditors is reduced to a negligible degree. The influence of suppliers changes from negative to positive. When the responsibilities of the five stakeholders are taken into account collectively, the influence of the government, media and employees remains positive, the effect of creditors diminishes, and the influence of suppliers shifts from a negative to a positive direction. After adding control factors, including the firm’s size and type, a more thorough explanatory model of corporate carbon disclosure was produced. The outcomes support this. The results indicate the following: Under complex political economy conditions, the government, the media, and employees have a positive correlation with the level of carbon disclosure; creditors have a negative correlation with it. The influence of suppliers is not significant. In summary, the results for government, media, and employees are more robust. They are key factors in promoting the quality of carbon information disclosure by enterprises. The effect of creditors may be related to the size of enterprises, and more specific policies need to be developed in the future depending on different enterprises. The influence mechanism of suppliers is more ambiguous, and empirical results confirm this. In other words, suppliers do not significantly affect this disclosure.

### 4.2. Recommendation

Combining the previous analysis and research findings, this paper makes the following recommendations to advance carbon information disclosure.

Government pressure can considerably boost the level of disclosure. Therefore, government policy guidance must be strengthened. Through tax policy regulation and the government’s guiding role, we can strengthen enterprises’ capacity to implement environmental protection transformation, low-carbon emission reduction. In this way, we help them switch from their old production mode of high emissions to a low-carbon and sustainable development mode. Enterprises will be considerably more incentivized to disclose carbon information when they have a set amount of money available for energy saving and emission reduction. In addition, whether it is taxation or tax support, the measurement basis is derived from carbon information, encouraging businesses to aggressively gather and arrange pertinent carbon information, laying the foundation for carbon information disclosure. As a result, there is a need to expand the role of government laws in pushing such disclosure. Investment in corporate environmental construction should be increased even further, environmental review processes should be improved, and the contribution of environmental performance to corporate value creation should be strengthened.

The shift in the direction of impact by creditors demonstrates that the starting point of the refinancing environmental verification policy is accurate. However, it needs to consider the different circumstances of different companies. This results in the impact of this policy not being positive. Therefore, while continuing to strengthen the constraints on creditors, it is also necessary to implement different measures for enterprises of different sizes and with different profitability. For instance, when it comes to financing rules, it should be prioritized to encourage the funding and development of projects in the environmental protection industries and offer these businesses greater loan options and concessions. Increased financing support should be offered to SMEs for technological innovation and low-carbon emission reduction. Green credit should be implemented to remove financing barriers standing in the way of the growth of a low-carbon economy. Simultaneously, appropriate punitive measures should be taken, such as deferring, limiting, or suspending loans, against enterprises that violate laws and regulations on environmental protection. At the same time, product and service innovation should be actively promoted to assist enterprises with energy conservation and environmental protection through various financing tools. We are actively developing financing tools such as medium-term notes, trust loans, financial leasing, and SME pooled notes to support our clients’ green projects. Preventing the environmental risks of enterprises by controlling their sources of funding is also a way to protect the rights of creditors. Creditors should include environmental risk assessment as an important part of their comprehensive risk assessment.

According to the empirical findings, media coverage can persuade companies to release more carbon information. Therefore, media publicity and supervision should be strengthened, and public opinion should play a driving role in such disclosure. As China is currently in an economic transition, the media’s monitoring role is frequently interfered with, and media freedom to report is limited. As a result, the state should put in place appropriate safeguards to ensure the security and freedom of the media industry. The media can also strengthen organizations’ monitoring power and influence by forming green alliances with other similar or related organizations, striving to spread low-carbon environmental awareness to the public while fully disclosing enterprises’ efforts and shortcomings in low-carbon energy conservation.

The level of carbon information disclosure increases with employee count. This suggests that as employees become more environmentally conscious, companies experience increasing pressure from their employees’ low-carbon demand. As a result, businesses should improve environmental training and publicity for their employees, popularize knowledge of environmental preservation from various perspectives, and promote employees’ understanding of ecological civilization. At the same time, employees must improve their sense of ownership and actively monitor the fulfillment of corporate social responsibility.

### 4.3. Research Limitations and Future Prospects

This study also has certain limitations, mainly reflected in the following two aspects.

This study employs text analysis to quantify carbon information from annual reports and CSR reports of firms because China has not yet created a carbon information database or a reliable grading agency. There is inevitably some subjectivity in the process of index construction. Additionally, some listed companies have not disclosed any carbon information, scoring 0 in the above framework, which may lead to some bias in the study results. With the continuous promotion of the policy, more and more businesses will try to share their carbon-related information. To further validate the driving variables and mechanisms of carbon information disclosure through more sample data, the next study could increase the sample size and the duration of the empirical investigation.

Secondly, the development of a low-carbon economy is uneven among regions [41], so further research should be carried out on grouping enterprises in different regions. Investigating whether the different stakeholders’ combined impact on corporate carbon disclosure is beneficial or detrimental is also worthwhile. The findings have significant implications for business environmental initiatives and government environmental governance.

## Figures and Tables

**Table 1:** Content of carbon information disclosure.

Variable	Explanation	References
CSR	Whether to publish a CSR report	
Risk	Whether to disclose fines related to environmental pollution Whether to disclose litigation related to environmental pollution	CDP ^1^
Strategy	Whether to set abatement plans, targets Whether there are management systems and institutions related to emission reduction Whether environmental training, awareness, and actions are conducted	CDSB ^2^
Governance	Whether to disclose the emission reduction of the “three wastes” Whether to disclose the treatment, recycling, and utilization of waste Whether to disclose the operation and renovation of environmental protection facilities Whether to disclose environmental capital investment, research, and innovation	PWC ^3^
Accounting	Whether to disclose greenhouse gas emissions Whether to disclose wastewater discharge Whether to disclose other solid emissions of pollutants	CDP ^1^
Performance and subsidies	Whether economic benefits from emission reductions are disclosed Whether there are honors related to environmental protection Whether there are environmental bonuses or subsidies	GRI ^4^
Validation/Authentication	Whether it has passed IS014001 environmental management system certification Whether it has been inspected by an independent third-party organization	PWC ^3^

^1^ Information from www.cdproject.net/en—US/Results/Pages/All—Investor—Reports.aspx (accessed on 11 October 2021. ^2^ Information from www.cdsb.net/ccrf/ClimateChangeReportingFramework(Edition1.1) (accessed on 11 October 2021). ^3^ Information from www.Pwc.com/gx/en/sustainability/publications/carbon-disclosure-project/downloads.jhtml (accessed on 14 October 2021). ^4^ Information from www.globalreporting.org/resourcelibrary/G3.1-Sustainability—Reporting—Guidelines (accessed on 14 October 2021).

**Table 2:** Full variable design.

Type	Codes	Description
Explained variables	CDI	Each secondary indicator is given the same weighting when scoring, with a range of 0–34 points
Explanatory variables	State	Percentage of state-owned shares
	Creditor	Gearing ratio
	Media	Natural logarithm of (1 + number of media reports)
	Staff	Natural logarithm of the number of employees
	SR	Top five suppliers’ purchases/total annual purchases
control variables	Industry	Priority emission units are assigned a value of 1, otherwise 0
	Size	Natural logarithm of total assets at the end of the year
	RA	Total return on assets

**Table 3:** Basic statistics on the quality of carbon information disclosure.

Year	Variable	Obs	Mean	Sd.	Min	Max
2017	CDI	118	3.1102	4.2342	0	17
2018	CDI	118	4.8220	4.6347	0	24
2019	CDI	118	5.3898	5.1454	0	27
2020	CDI	118	5.6017	4.9061	0	24
2021	CDI	118	7.6780	5.6627	0	27
Total	CDI	590	5.3203	5.1491	0	27

**Table 4:** Variable correlation coefficient matrix.

	CDI	State	Creditor	Media	Staff	SR	Industry	Size	RA
**CDI**	1								
**State**	0.333 ***	1							
**Creditor**	0.329 ***	0.309 ***	1						
**Media**	0.483 ***	0.309 ***	0.390 ***	1					
**Staff**	0.634 ***	0.368 ***	0.491 ***	0.598 ***	1				
**SR**	−0.114 ***	−0.136 ***	−0.212 ***	−0.189 ***	−0.344 ***	1			
**Industry**	0.641 ***	0.252 ***	0.411 ***	0.326 ***	0.413 ***	−0.024	1		
**Size**	0.660 ***	0.442 ***	0.534 ***	0.649 ***	0.920 ***	−0.277 ***	0.446 ***	1	
**RA**	−0.337 ***	−0.341 ***	−0.527 ***	−0.272 ***	−0.386 ***	−0.041	−0.468 ***	−0.469 ***	1

Significance levels of 1% is denoted by ***.

**Table 5:** Multi-collinearity discriminant table.

Variable	VIF	1/VIF
Size	8.54	0.117076
Staff	7.11	0.140604
Media	1.76	0.569473
Creditor	1.69	0.590786
RA	1.69	0.591324
Industry	1.45	0.690247
State	1.30	0.770244
Supplier	1.19	0.840036
Mean VIF	3.09	

**Table 6:** Multiple regression results.

	(1)	(2)	(3)	(4)	(5)	(6)	(7)
VARIABLES	CDI	CDI	CDI	CDI	CDI	CDI	CDI
State	0.333 ***					0.103 ***	0.051 *
	(8.57)					(3.03)	(1.74)
Creditor		0.329 ***				−0.006	−0.123 ***
		(8.44)				(−0.17)	(−3.66)
Media			0.483 ***			0.145 ***	0.068 **
			(13.36)			(3.69)	(1.98)
Staff				0.634 ***		0.553 ***	0.184 ***
				(19.90)		(12.76)	(2.67)
Supplier					−0.114 ***	0.116 ***	0.042
					(−2.78)	(3.52)	(1.48)
Industry							0.945 ***
							(15.20)
Size							0.327 ***
							(4.34)
RA							0.077 **
							(2.30)
Constant	0.000	0.000	0.000	0.000	0.000	0.000	−0.444 ***
	(0.00)	(0.00)	(0.00)	(0.00)	(0.00)	(0.00)	(−11.39)
Observations	590	590	590	590	590	590	590
R-squared	0.111	0.108	0.233	0.402	0.013	0.440	0.613
r2_a	0.109	0.106	0.232	0.401	0.0113	0.435	0.608
F	73.37	71.16	178.6	395.9	7.723	91.62	115.3

Robust t-statistics in parentheses; *** *p* < 0.01, ** *p* < 0.05, * *p* < 0.1.

**Table 7:** Robustness test results (after tailing process).

	(1)	(2)	(3)	(4)	(5)	(6)	(7)
VARIABLES	CDI	CDI	CDI	CDI	CDI	CDI	CDI
State	0.331 ***					0.097 ***	0.054 *
	(8.57)					(2.89)	(1.90)
Creditor		0.329 ***				−0.007	−0.122 ***
		(8.54)				(−0.19)	(−3.65)
Media			0.490 ***			0.134 ***	0.067 *
			(13.48)			(3.32)	(1.94)
Staff				0.642 ***		0.564 ***	0.200 ***
				(20.19)		(12.79)	(2.94)
Supplier					−0.113 ***	0.112 ***	0.049 *
					(−2.78)	(3.43)	(1.77)
Industry							0.944 ***
							(15.48)
Size							0.317 ***
							(4.26)
RA							0.085 **
							(2.39)
Constant	−0.003	−0.004	−0.007	−0.006	−0.004	−0.006	−0.446 ***
	(−0.08)	(−0.09)	(−0.19)	(−0.21)	(−0.10)	(−0.21)	(−11.70)
Observations	590	590	590	590	590	590	590
R-squared	0.111	0.110	0.236	0.409	0.013	0.442	0.620
r2_a	0.110	0.109	0.235	0.408	0.0113	0.437	0.615
F	73.46	72.86	181.6	407.6	7.709	92.38	118.7

Robust t-statistics in parentheses; *** *p* < 0.01, ** *p* < 0.05, * *p* < 0.1.

**Table 8:** Robustness test results (change in variable design).

	(1)	(2)	(3)	(4)	(5)	(6)	(7)
VARIABLES	CDI	CDI	CDI	CDI	CDI	CDI	CDI
State	0.333 ***					0.103 ***	0.059 *
	(8.57)					(3.03)	(1.95)
Creditor		0.329 ***				−0.006	−0.147 ***
		(8.44)				(−0.17)	(−4.27)
Media			0.483 ***			0.145 ***	0.103 ***
			(13.36)			(3.69)	(3.03)
Staff				0.634 ***		0.553 ***	0.348 ***
				(19.90)		(12.76)	(4.58)
Supplier					−0.114 ***	0.116 ***	0.046
					(−2.78)	(3.52)	(1.62)
Industry							0.914 ***
							(14.88)
Size							0.111
							(1.34)
ROE							−0.028
							(−0.99)
Constant	0.000	0.000	0.000	0.000	0.000	0.000	−0.429 ***
	(0.00)	(0.00)	(0.00)	(0.00)	(0.00)	(0.00)	(−11.02)
Observations	590	590	590	590	590	590	590
R-squared	0.111	0.108	0.233	0.402	0.013	0.440	0.601
r2_a	0.109	0.106	0.232	0.401	0.0113	0.435	0.596
F	73.37	71.16	178.6	395.9	7.723	91.62	109.4

Robust t-statistics in parentheses; *** *p* < 0.01, * *p* < 0.1.

## Data Availability

The financial data and basic information data from the companies are obtained from the CSMAR database, see http://www.gtarsc.com/, accessed on 1 July 2022. The media attention data are obtained from the CNRDS database, see www.cnrds.com, accesses on 6 July 2022.
